# Assessing Impacts on Unplanned Hospitalisations of Care Quality and Access Using a Structural Equation Method: With a Case Study of Diabetes

**DOI:** 10.3390/ijerph13090870

**Published:** 2016-09-01

**Authors:** Peter Congdon

**Affiliations:** School of Geography and Life Sciences Institute, Queen Mary University of London, London E1 4NS, UK; p.congdon@qmul.ac.uk; Tel.: +44-(0)-20-7882-8200

**Keywords:** quality of care, diabetes, access, structural equation model, emergency admissions, deprivation

## Abstract

*Background*: Enhanced quality of care and improved access are central to effective primary care management of long term conditions. However, research evidence is inconclusive in establishing a link between quality of primary care, or access, and adverse outcomes, such as unplanned hospitalisation. *Methods*: This paper proposes a structural equation model for quality and access as latent variables affecting adverse outcomes, such as unplanned hospitalisations. In a case study application, quality of care (QOC) is defined in relation to diabetes, and the aim is to assess impacts of care quality and access on unplanned hospital admissions for diabetes, while allowing also for socio-economic deprivation, diabetes morbidity, and supply effects. The study involves 90 general practitioner (GP) practices in two London Clinical Commissioning Groups, using clinical quality of care indicators, and patient survey data on perceived access. *Results*: As a single predictor, quality of care has a significant negative impact on emergency admissions, and this significant effect remains when socio-economic deprivation and morbidity are allowed. In a full structural equation model including access, the probability that QOC negatively impacts on unplanned admissions exceeds 0.9. Furthermore, poor access is linked to deprivation, diminished QOC, and larger list sizes. *Conclusions*: Using a Bayesian inference methodology, the evidence from the analysis is weighted towards negative impacts of higher primary care quality and improved access on unplanned admissions. The methodology of the paper is potentially applicable to other long term conditions, and relevant when care quality and access cannot be measured directly and are better regarded as latent variables.

## 1. Introduction

Improving quality of, and access to, primary care are major strategic foci in transforming primary care in the face of growing demand and the increasing burden of long term conditions [1]. For example, in the English National Health Service (NHS), a primary care incentive scheme known as the Quality Outcomes Framework (or QOF) was introduced in 2004/2005 to promote improved chronic disease management [2]. The impact of this scheme on performance and care quality is measured by a set of indicators within various clinical domains (disease types or groupings).

Although findings are not consistent, quality of primary care may reduce unplanned emergency hospital admissions, which have major cost implications [3,4,5]. There is debate about impacts on adverse outcomes (e.g., premature mortality, avoidable hospitalisations) of quality as measured by the QOF indicators [6,7].

Unplanned admissions may also be reduced by improved access [8]. Access to care can be defined in various ways [9,10]. Utilisation-based measures of access provide a partial picture [11], and many UK studies now focus on patient-reported access to primary care, namely patient experience or perception of access: how easy patients believe it is to access their GP practice [12]. This includes factors such as convenience of GP surgery opening hours and ease of making appointments [13]. Policies to improve access to health care have included schemes to extend operating hours in GP practices, including GP appointments in the evenings or at weekends, measures to reduce inequity in access, and a target that every patient should be able to see a GP within 48 h [14,15].

Perceived access and quality of care are interrelated as higher patient satisfaction and engagement are linked to better self-care and clinical outcomes [16,17]. Indeed patient experience can be conceived as an aspect of quality of care, with the 2008 NHS Next Stage Review identifying patient experience as one dimension of quality, together with clinical effectiveness and patient safety [18]. More specifically in terms of avoidable hospitalisations, recent studies (e.g., [19]) find a negative relation between such hospitalisations and improved access, as measured by responses to the GP Patient Survey.

Impacts of primary quality of care and access on adverse outcomes, such as emergency admissions, may, however, be confounded by socioeconomic differences and health behaviours [20,21,22]. Thus, [23] mention that positive associations between ambulatory sensitive admissions and area deprivation may be related to socio-economic inequalities in health care provision; for example, see [24] regarding diabetes.

Impacts of deprivation on adverse outcomes may be partly mediated by variations in quality of care and access linked to socioeconomic deprivation. Thus, [25] found that poorly performing practices (in terms of diabetes care) tended to have deprived populations, and variations in access have been linked to characteristics of patient populations [11]. Links between access and deprivation may be partly an indirect expression of inequities in the supply of GPs with several studies showing mismatch between GP supply and health need (e.g., [26]).

Impacts of quality of care and access, as measured in regression or other statistical analysis, may also be affected by the methods used to derive summary indices. Quality of care and access cannot be measured by any single indicator and may, instead, be better regarded as latent quantities indirectly measured by a set of manifest observed indicators. Whereas the official method used to obtain quality scores under the QOF scheme does not adjust for overlapping correlation between input variables, multivariate techniques, such as factor analysis and structural equation methods, may seek instead to derive scores adjusted for multicollinearity [27,28].

The particular focus in the present paper is on the measurement of quality of care for diabetes, and assessing impacts of care quality and access on diabetes related emergency admissions. A factor score (latent indicator) method for measuring care quality and perceived access is proposed, with impacts on diabetes related emergency admissions assessed as part of a broader structural equation model (SEM). Manifest indicators of diabetes care (from which the latent indicator is derived) are taken from the Quality Outcomes Framework, while manifest access indicators are from a national survey of patients. A Bayesian inference approach is adopted, using Markov Chain Monte Carlo (MCMC) sampling [29], via the rjags program in R [30,31].

## 2. Case Study

A case study involves 90 GP practices in two London Clinical Commissioning Groups (Havering CCG, and Barking and Dagenham CCG), which are agencies coordinating provision of primary care. Patient populations are drawn predominantly from two north London boroughs—Havering, and Barking and Dagenham. Around 96% of the residents of these two boroughs have primary care provided by GP practices affiliated to the two CCGs.

As observed measures of care quality, the study uses four clinical indicators of diabetes care from the Quality Outcomes Framework for the financial year 2013–2014. The choice of indicators (see Table 1) is motivated by relevance in predicting risk of hospitalisation or complication, and also to avoid the duplication present in the official QOF indicators, such as overlapping measures of HbA1c and blood pressure.

Regarding blood pressure, cholesterol, and blood glucose control (the first three indicators in Table 1), several studies confirm their importance in preventing complications and hospitalisation [21,32,33]. The National Audit Office [34] (Section 2.9) mentions that “the risk of developing diabetic complications can be minimised by early detection and management of high levels of blood glucose (measured using HbA1c), blood pressure and cholesterol”. Regarding influenza immunisation, U.S. data [35] show patients with diabetes are six times more likely to be hospitalized from complications of influenza or pneumonia than those in the general population, while a UK study [36] demonstrated that influenza vaccination significantly reduced hospital admissions by diabetic patients.

As discussed in the Introduction, access to primary care is defined in terms of patient experience or perception of access, as drawn from NHS Patient Surveys of primary care experience and satisfaction. The indicators used in the present study (see Table 1) are from the 2014 patient survey and refer to patients (a) dissatisfied with surgery opening hours; (b) stating that appointments were not convenient; (c) stating their overall experience of the surgery as poor; and (d) reporting that waiting times at surgery were too long.

Studies such as [13] find that access to care may depend on GP supply or GP practice list size. These are incorporated in the analysis as causes of, as opposed to indicators of, varying access—following the multiple indicators-multiple causes terminology in the SEM literature (e.g., [37]).

As well as deriving composite quality and access scores, we seek to assess how far quality of care and access affect emergency hospital admissions by GP practices in 2013/2014, where diabetes is among the contributing diagnoses, either as the primary diagnosis or as the leading subsidiary diagnosis [38,39].

Hospital use may be affected by morbidity and demographic factors, including variations in socio-economic status [40]. Levels of diabetes morbidity are measured by practice level age standardised rates for diagnosed diabetes. Regarding socio-economic conditions, the study region shows wide differences: 10 of its 260 neighbourhoods (Census lower super output areas, abbreviated as LSOAs) are in the most affluent decile of such neighbourhoods across England, while at the other extreme, 13 neighbourhoods are among the most deprived 10% of all English neighbourhoods.

## 3. Methods

Some studies have used official QOF achievement scores (intended to measure quality of care) in assessing impacts on patient outcomes, for example in regression analysis of emergency admission rates [41,42]. However, these scores do not adjust for overlapping correlations between the input indicators used to obtain overall achievement scores within clinical domains such as diabetes. The official scores also take percentage indicators of achievement as known without error, regardless of the size of the patient population denominator (which may vary considerably between indicators and GP practices) and, therefore, do not allow for sampling variability (i.e., varying precision) in the percentage indicators based on binomial data.

### 3.1. Quality Scores in a Structural Equation Model

We wish to take account of sampling variability and control for correlations between measured input indicators, and obtain a composite measure of care quality with loadings on input indicators which are optimal in relation to potential outcomes (e.g., emergency admissions). For example, some indicators may have greater impact on emergency admissions.

Accordingly we define a structural equation model with a measurement model in which quality of care as a latent variable, denoted F, is measured by a set of observed input indicators Z, and an outcome model relating diabetes related emergency admissions Y to quality of care F (see Appendix A for a formal development). There are I = 90 GP practices and J = 4 manifest (observed) clinical indicators, Z_ij_ (i = 1, …, I; j = 1, …, J), to define the latent quality scale. These are in the form of patient totals achieving a given performance target, and are from the Quality Outcomes Framework for 2013–2014.

We wish to represent stochastic uncertainty in the clinical indicators. Thus, let Z_ij_ denote the number of diabetic patients in practice i for whom a particular clinical threshold is attained (e.g., diabetic patients with blood pressure reading below 150/90 mmHg), and N_ij_ denote the relevant patient denominator (e.g., number of registered diabetes patients). A binomial likelihood model with unknown performance attainment probabilities is adopted for the observations (Z_ij_, N_ij_) (see Appendix A), with the likelihood allowing for overdispersion.

It is assumed that variability in performance attainment probabilities is explained by normally-distributed latent quality scores F_i_. Regarding these scores, alternative distribution assumptions might be considered, for example, assuming F_i_ to be Student’s t-distributed rather than as normal. This might be relevant if there were distinct outlier practices with unduly high/low QOC or access.

The goal is not only to summarize quality of care in a composite index, but assess how far quality of care affects adverse hospital outcomes. In principle there may be more than one such outcome, but here there is a single outcome, the total of diabetes related emergency admissions, Y_i_. In the baseline model (model 1) relative risks of emergency admission ν_i_ are predicted from a log-link regression including only quality of care:

log(ν_i_) = γ_0_ + γ_1_F_i_ (1)

As F_i_ is a positive measure of quality, one would expect γ_1_ to be significantly negative if high QOC significantly reduces unplanned admissions. From the Bayesian estimation process, one can the estimated probability Pr(γ_1_ < 0|Y,Z) that the coefficient is negative. This provides an alternative method of assessing the direction of effect to the 95% credible interval [43].

A second model (model 2) allows for the impact on emergency admissions of a score measuring area socioeconomic deprivation, D_i_. This is the 2015 index of income deprivation [44], calculated for GP practice populations. This is used instead of the main Index of Multiple Deprivation (IMD), which includes standardised emergency admission rates in the constituent variables [40].

A subsidiary analysis, not reported on in detail, assessed sensitivity to deprivation impacts of using the employment deprivation score [44] rather than income deprivation, throughout models 2 and 3, as considered below. This shows a slight diminution in goodness of fit as the employment domain had a lower impact on emergency diabetes admissions.

We further consider the impact on emergency admissions of diabetes morbidity (M_i_), namely, the age-standardised rate at mid-2013 of diagnosed diabetes—see Equation (A8). Model 2 also extends the model for the quality scores so that deprivation can also potentially affect quality of care, as indicated by several studies [45,46,47].

### 3.2. Access to Care

As mentioned above, unplanned hospitalisation may also be linked to various aspects of access to primary care. In common with a number of UK recent studies, perceived patient access to primary care is based on indicators from the GP Patient Survey [48]. Just as for quality of care, access is conceived as a latent quality, not measurable by any single indicator.

Thus, the structural equation model (model 3) now has a measurement model in which quality of care as a latent variable F is measured by a set of observed input indicators Z, another measurement model in which poor access is a latent variable G measured by a set of observed indicators W, and an outcome model relating diabetes related emergency admissions Y to quality of care F and access G.

Let W_ik_ (k = 1, …, K) be patient totals rating access as poor, and M_ik_ be denominator populations (i.e., all patients surveyed, regardless of their rating). The indicators used are as in Table 1. Since access and quality of care may potentially be interrelated [13], it is assumed that F_i_ and G_i_ are bivariate normal, with covariance ∑ and correlation ρ.

Furthermore, we allow care quality and care access to both depend on deprivation (e.g., [49]), with respective coefficients β_1_ and β_2_. Access to care may also depend on GP supply, namely full time equivalent GPs per 1000 patients, S_1i_, and GP practice list size, S_2i_ [13]. These are included as additional potential causal influences on access, with respective coefficients β_3_ and β_4_; see Equation (A20).

## 4. Analysis and Results

Analyses are carried out using the rjags package in R, with detailed assumptions, model checks and fit measures described in Appendix B. Table 2 shows satisfactory predictive checks for all three models, all between 0.1 and 0.9. The WAIC fit criteria (Appendix B) show a gain in moving from model 1 to model 2, but no change in fit between models 2 and 3.

### 4.1. Regression Findings

Table 3 shows estimated regression coefficients under the three models for the emergency admissions regression. Table 4 sets out summaries for λ_j_ and κ_k_, the loadings in the measurement model(s) for care quality and care access, together with β coefficients relating these constructs to deprivation and supply variables.

Table 3 shows that under model 1, the impact of care quality on emergency admissions has 95% credible interval (−0.50, 0.01) [50] (p. 1063). casts doubt on converting such an interval (which just straddles zero) into evidence for a null effect. Under a Bayesian approach, whether quality has a negative impact can be assessed via the posterior probability Pr(γ_1_ < 0|Y,Z) [43] (Chapter 5). We find that there is a 97% probability that the coefficient is negative.

The measurement model coefficients (Table 4) for model 1 show broadly similar relevance of each of the observed indicators (see Table 1) in defining quality of care. Posterior means on the loadings λ_j_ range from 0.59 to 1.29.

Under model 2, the impacts of deprivation and morbidity on emergency admissions are both highly significant. This model also allows part of the impact of deprivation to be indirect, in that quality of care depends on deprivation; see Equation (A12) in Appendix A. In fact, this indirect effect is negative, as expected, but not pronounced: we find a probability of 0.73 that Pr(β_1_ < 0|Y,Z), where a negative coefficient β_1_ means that higher practice deprivation is associated with lower quality of care.

**Figure 1:**
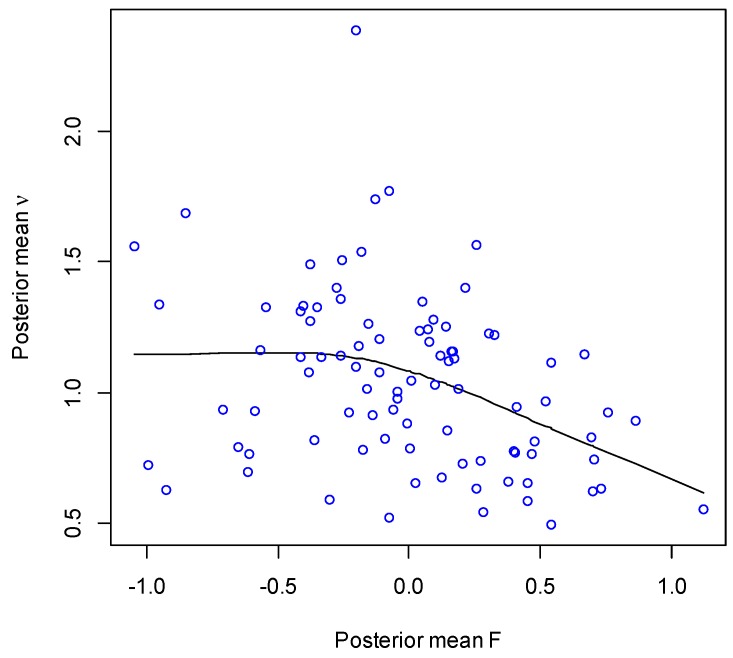
Quality of care and emergency admission relative risk.

The impact of care quality itself on emergency admissions remains significant in model 2, as assessed by a probability Pr(γ_1_ < 0|Y,Z) of 0.974. Figure 1 plots the posterior means (under model 2) of the quality score F_i_ against those for emergency admission relative risk ν_i_, together with a LOWESS plot (locally weighted scatterplot smoothing). This plot suggests a threshold effect, with greater impacts of high care quality in reducing emergency admissions.

Model 3, the full structural model, involves access as well as quality of care. Models 2 and 3 have very similar fit measures, and statistical criteria may be supplemented by substantive considerations, since on the basis of accumulated evidence from other studies, model 3 may be regarded as providing a more complete description of the interrelated processes. Regarding the measurement model for access, the highest loading κ_k_ (with posterior mean approaching 2) is for the variable overall poor experience of the surgery.

Of particular interest in the estimates for model 3 are positive impacts of deprivation on perceived poor access to care (the β_2_ coefficient), and a negative correlation ρ between poor access and quality of care. The respective 95% credible intervals are (0.23, 0.68) and (−0.61, −0.12). There is also a significant positive impact on poor access of larger GP list sizes (the population total served by a GP practice), and a mostly negative impact of GP supply, with 95% interval (−0.82, 0.07). A list size effect on access (i.e., perceived access better in small practices) is also reported by [13].

**Figure 2:**
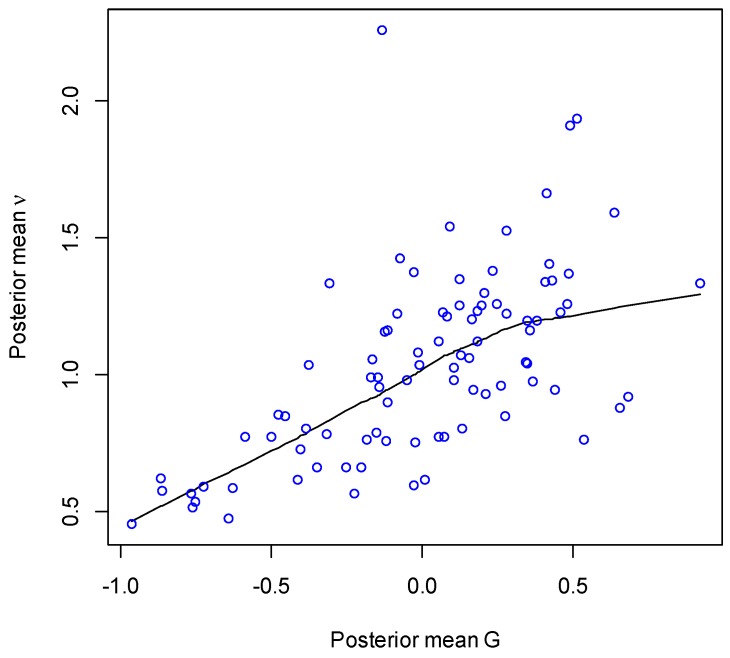
Poor access and emergency admission relative risk.

The impact of poor access on emergency admissions has a posterior density concentrated on positive values, with a 95% credible interval (−0.10, 0.46), and a 90% probability of a positive effect. Figure 2 plots the posterior means of the poor access score G_i_ against those for emergency admission relative risk ν_i_, with LOWESS smooth included.

**Figure 3:**
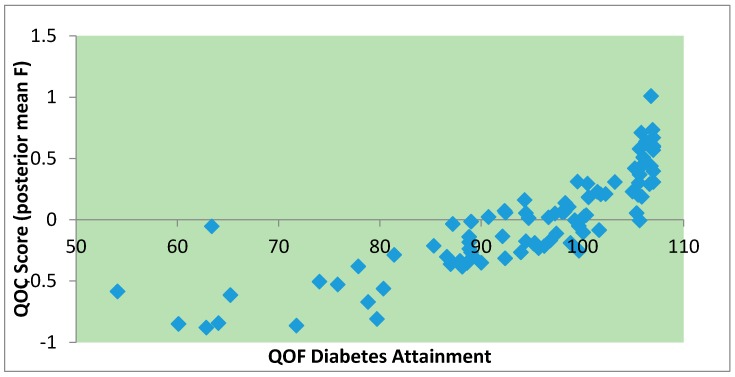
Modelled quality of care scores vs. QOF attainment scores.

It is of interest to assess how the QOC scores (the F_i_) compare with the official diabetes QOF attainment scores. The correlation between the posterior mean F from model 3 and the QOF attainment scores is 0.84, and their interrelationship is shown in Figure 3. It is apparent that the modelled QOC scores provide extra discrimination in measuring quality of care at the highest levels of the official attainment score, namely, 23 practices with official attainment scores between 105 and the maximum possible 107.

### 4.2. Sensitivity Analysis

The above analysis may be affected by distributional assumptions. The derivation of latent factors for care quality and access is central to the proposed approach, and so effects were assessed of adopting a Student’s t distribution of factor scores rather than a normal density, as this heavier tailed density may be more robust to any outlier practices with unduly high/low quality of care or access.

A bivariate Student’s t density for the factor scores in model 3 was achieved using a scale mixture approach [51] (p. 138), with a default degrees of freedom set at four, as advocated by [52] (p. 449). This provides a fourth model, results from which are also included in Table 2, Table 3 and Table 4. This shows no marked change in fit, but slightly enhances the impact of quality of care on emergency admissions, with a 93% probability that the impact is negative.

### 4.3. Implications for Geographic Differences in Access

As noted above, around 96% of residents in the case study region have their primary care provided by GP practices in the two CCGs (which have the same names as the boroughs, namely Barking and Dagenham CCG, and Havering CCG). Hence, GP practice differences in access and care quality (measured by the G and F scores) can be translated into implied geographic differences in access and care quality. These are obtained using cross-reference files of populations classified both by LSOA and by GP practice (hence of dimension 260 by 90). LSOA weighted averages for access or care quality are based on proportions of each neighbourhood population cared for by each of the 90 GP practices.

**Figure 4:**
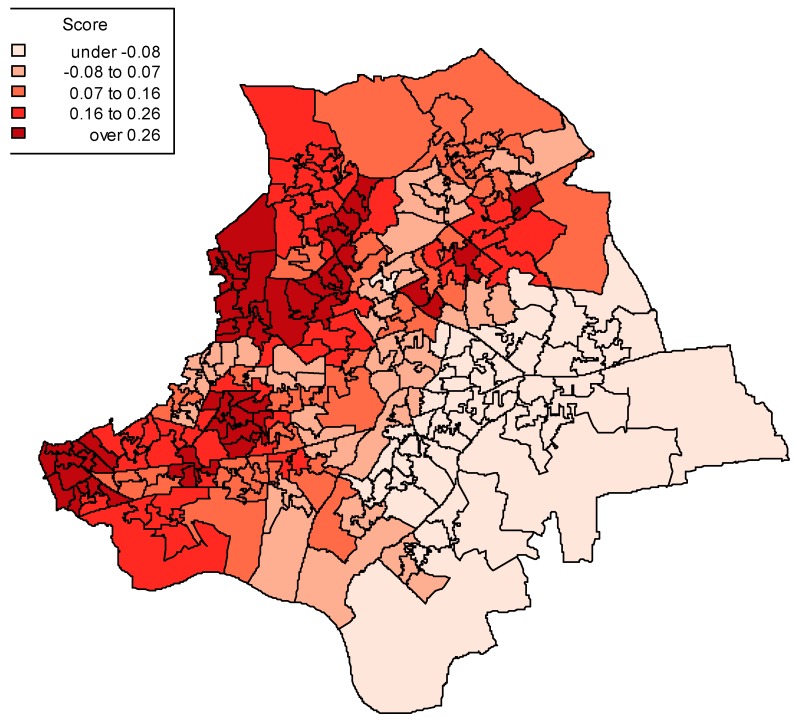
LSOA averages on posterior mean access scores, model 3.

**Figure 5:**
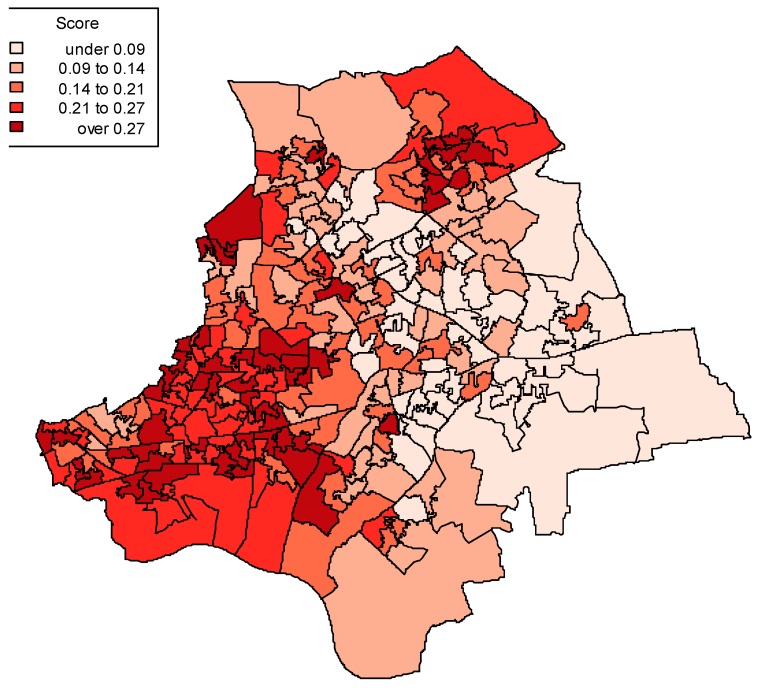
LSOA income deprivation scores.

Figure 4 accordingly maps out access scores for the 260 neighbourhoods (LSOAs) using the posterior mean G scores for GP practices, and shows clear geographic clustering of poor access. An accompanying map (Figure 5) shows LSOA scores for income deprivation, and associations between the geographic patterns of deprivation and poor access are apparent.

## 5. Conclusions

This paper has developed a structural equation model for quality of primary care and perceived access to primary care, and assessed their impacts on unplanned hospital admissions for diabetes, while allowing also for socio-economic deprivation, morbidity, and supply indicators. This methodology is potentially applicable to other long term conditions, and relevant when care quality and access cannot be measured directly and are better regarded as latent variables. Methodology for measuring quality of care and access may affect findings regarding their impacts on outcomes, such as unplanned admissions.

In that regard, a number of studies report insignificant or negligible effects of quality of care on adverse hospital outcomes, such as avoidable or ambulatory sensitive hospital admissions. For example [22] report small associations between official QOF scores and emergency admissions, whilst impacts of socio-economic deprivation were much stronger. [40] also obtain a strong effect of deprivation on potentially avoidable emergency admissions, and mention that this is in part because deprivation is correlated with morbidity.

In the case study region of the current paper, primary care population register data for diagnosed diabetes (standardized prevalence rates in 2013) show wide variation between GP practices, and there is a 0.70 correlation between diabetes prevalence and income deprivation. This inter-correlation is controlled for in models 2 and 3, but a strong deprivation effect remains.

The analysis also suggests a strong indirect effect of deprivation on emergency admissions through access, though not a strong indirect impact through quality of care. The positive association between income deprivation and perceived poor access under model 3 can be represented in terms of implied neighbourhood access variations (as shown by Figure 4). This pattern is consistent with evidence that care access in deprived, as compared to affluent areas, may be related to variations in primary care provision, especially provision that matches health care need, with continuing evidence of an inverse care law [53,54].

Some potential extensions of the model framework and analysis of this paper may be proposed. Thus, an additional dimension that has been shown in the literature to have a substantial effect on quality of care is continuity of care, “in the sense of a patient repeatedly consulting the same doctor and forming a therapeutic relationship” [55]. For example, [56] in a Korean study derive different indices of continuity from detailed patient consultation histories. One could potentially form a composite index of continuity based on combining different indices, but a UK application is impeded by non-availability of patient primary care consultation data.

Interpretation of quality and access effects on adverse outcomes has preoccupied several recent studies. After accounting for the impact of deprivation and morbidity in model 2—which fits as well as the full model to the hospitalisation and quality indicators—the effect of quality of care on unplanned admissions remains significant: a 97% probability for a negative impact. For the full model, quality of care has a 91.8% probability of a negative impact, and poor access has a 90% probability of a positive impact. The latter two impacts are increased slightly under model 4 with a bivariate Student’s t model for correlated quality and access.

These probabilities may be interpreted from a Bayesian perspective in terms of marginal Bayes factors [57]. The prior on the regression coefficients is neutral with regard to the direction (positive or negative) of the coefficient, so a 91.8% posterior probability of a negative impact of quality on emergency admissions implies a marginal Bayes factor of 11.2. Using standard guidelines [58], this counts as strong evidence of an effect.

In such terms, the analysis of the present study is not consistent with a null finding regarding impacts of quality of care and access on potentially avoidable emergency admissions. Rather the regression evidence is weighted towards negative impacts of higher primary care quality and improved access on unplanned admissions. Other aspects of the model are also important in healthcare terms, such as poor access being greater for deprived practice populations.

## Figures and Tables

**Table 1:** Indicators for quality of care and access.

Concept	Indicator	Numerator	Denominator	Overall Percent Rate	10th Percentile of Practice Rates	90th Percentile of Practice Rates
Quality	Z_1_	Patients with last blood pressure reading of 150/90 mmHg or less	Diagnosed diabetes patients	92.1	85.8	97.2
	Z_2_	Patients whose last measured total cholesterol 9 of 5 mmol/L or less	Diagnosed diabetes patients	79.4	70.2	87.7
	Z_3_	Patients with last IFCC-HbA1c of 64 mmol/mol or less	Diagnosed diabetes patients	74.6	61.7	85.6
	Z_4_	Patients with influenza immunization in most recent winter period.	Diagnosed diabetes patients	93.0	84.3	99.4
Access	W_1_	Dissatisfied with surgery opening hours	Surveyed Patients at Practice	12.6	4.6	19.1
	W_2_	Last appointment not very convenient or not at all convenient	Surveyed Patients at Practice	9.7	3.0	17.3
	W_3_	Overall experience of making an appointment fairly poor or very poor	Surveyed Patients at Practice	13.1	1.9	23.9
	W_4_	Waiting times at surgery too long	Surveyed Patients at Practice	13.4	2.7	29.5

**Table 2:** Model checks and fit measures.

Variable Type	Description	Posterior Predictive Checks			
		Model 1	Model 2	Model 3	Model 4
Patient outcome (Y)	Diabetes related emergency admissions	0.42	0.23	0.18	0.19
Clinical Quality Indicators (Z variables)	Last blood pressure reading 150/90 mmHg or less	0.53	0.54	0.53	0.53
	Last total cholesterol 5 mmol/L or less	0.56	0.56	0.57	0.56
	Last HbA1c 64 mmol/mol or less	0.62	0.62	0.63	0.60
	Influenza immunization, most recent winter	0.51	0.51	0.52	0.51
Access Indicators (W variables)	Dissatisfied with opening hours			0.62	0.61
	Appointments not convenient			0.89	0.87
	Overall experience of surgery poor			0.82	0.82
	Waiting times at surgery too long			0.57	0.58
		Fit (WAIC) *			
		**Model 1**	**Model 2**	**Model 3**	**Model 4**
	Emergency admissions	530.0	501.3	501.5	502.8
	Clinical indicators	2248.4	2247.0	2246.3	2248.7
	Access Indicators			1684.4	1685.5

* Lower value for better fit.

**Table 3:** Coefficients for outcome model (emergency diabetes related admissions).

	Symbol	Posterior Summary					
		Mean	St. Devn.	2.5%	97.5%	Probability Regression Coefficient Positive	Probability Regression Coefficient Negative
Model 1							
Intercept	γ_0_	0.02	0.05	−0.09	0.12	0.624	0.376
Quality of Care	γ_1_	−0.25	0.13	−0.50	0.01	0.029	0.971
Overdispersion parameter	α	0.14	0.04	0.07	0.23		
Model 2							
Intercept	γ_0_	−0.02	0.04	−0.10	0.06	0.342	0.658
Quality of Care	γ_1_	−0.20	0.10	−0.41	0.00	0.026	0.974
Deprivation	γ_2_	0.31	0.12	0.07	0.56	0.995	0.005
Diabetes morbidity	γ_3_	0.09	0.03	0.04	0.14	0.999	0.001
Overdispersion parameter	α	0.04	0.03	0.00	0.10		
Model 3							
Intercept	γ_0_	−0.03	0.04	−0.12	0.05	0.217	0.783
Quality of Care	γ_1_	−0.17	0.12	−0.42	0.07	0.082	0.918
Deprivation	γ_2_	0.24	0.13	−0.01	0.50	0.967	0.033
Poor Access	γ_3_	0.18	0.14	−0.10	0.46	0.900	0.101
Diabetes morbidity	γ_4_	0.09	0.02	0.04	0.14	1.000	0.000
Overdispersion parameter	α	0.03	0.03	0.00	0.10		
Model 4							
Intercept	γ_0_	−0.03	0.04	−0.11	0.06	0.278	0.722
Quality of Care	γ_1_	−0.17	0.12	−0.41	0.06	0.070	0.930
Deprivation	γ_2_	0.25	0.13	−0.01	0.51	0.969	0.031
Poor Access	γ_3_	0.18	0.14	−0.09	0.46	0.909	0.091
Diabetes morbidity	γ_4_	0.09	0.03	0.04	0.14	0.999	0.001
Overdispersion parameter	α	0.03	0.03	0.00	0.09		

**Table 4:** Measurement model parameters.

	Symbol	Posterior Summary											
		Model 1			Model 2			Model 3			Model 4		
		Mean	2.5%	97.5%	Mean	2.5%	97.5%	Mean	2.5%	97.5%	Mean	2.5%	97.5%
*Clinical Quality*													
Blood pressure ≤ 150/90 mmHg	λ_1_	1			1			1			1		
Total cholesterol 5 mmol/L or less	λ_2_	0.59	0.39	0.83	0.57	0.37	0.82	0.63	0.41	0.88	0.61	0.39	0.89
Last HbA1c 64 mmol/mol or less	λ_3_	0.74	0.51	1.00	0.70	0.49	0.96	0.77	0.53	1.04	0.75	0.51	1.04
Influenza immunization, most recent winter	λ_4_	1.29	0.68	1.92	1.26	0.67	1.86	1.33	0.76	1.94	1.26	0.69	1.93
*Poor Access*													
Dissatisfied with opening hours	κ_1_							1			1		
Appointments not convenient	κ_2_							1.35	0.91	1.90	1.29	0.91	1.71
Overall experience of surgery poor	κ_3_							1.89	1.39	2.54	1.87	1.34	2.45
Waiting times at surgery too long	κ_4_							1.47	0.95	2.11	1.36	0.87	1.90
*Correlation between constructs*	ρ							−0.38	−0.61	−0.12	−0.34	−0.58	−0.07
*Impacts of Deprivation*													
On quality of care	β_1_				−0.09	−0.38	0.20	−0.08	−0.35	0.19	−0.11	−0.45	0.23
On poor access	β_2_							0.45	0.23	0.68	0.45	0.24	0.69
*Impacts of Practice Factors on Poor Access*													
GP Supply (FTE GPs per 1000 Patients)	β_3_							−0.37	−0.82	0.07	−0.36	−0.85	0.09
List Size (in 000s)	β_4_							0.053	0.022	0.087	0.047	0.014	0.079

## Appendix A. Formal Aspects of Statistical Models

For quality of care indices, let Z_ij_ denote the number of diabetic patients in practice i for whom a particular clinical threshold is attained (e.g., diabetic patients with blood pressure reading below 150/90 mmHg), and N_ij_ denote the relevant patient denominator (e.g., number of registered diabetes patients). One has binomial sampling:

Z_ij_ ~ Bin(N_ij_,π_ij_), j = 1, …, J (A1)

with performance attainment probabilities π_ij_ that are unknown parameters to be estimated.

In the simplest model (model 1) it is assumed that variability in attainment probabilities π_ij_ is explained by a normally-distributed common quality factor F_i_, and random residual effects u_ij_, accounting for over-dispersion, which are also normally distributed. Thus with a logit link, and intercepts ς_j_, one has:

logit(π_ij_) = ς_j_ + λ_j_F_i_ + u_ij_, (A2)

u_ij_ ~ N(0,ψ_j_), (A3)

F_i_ ~ N(0,θ). (A4)

For identifiability, the loading λ_1_ = 1 and so the variance θ of the factor scores is an unknown [59]. The remaining loadings are constrained to be positive in line with a quality of care interpretation for the F scores, and for computational stability, to avoid label switching during MCMC updating.

In model 1, hospitalisation risks are also related to the latent QOC score. Denoting observed emergency admissions as Y_i_ and expected admissions as E_i_, it is assumed that the Y_i_ are negative binomial, with overdispersion parameter α, Y_i_ ~ NB(E_i_ν_i_,α), and likelihood:

(A5) $${\left(\frac{1}{1+\alpha \mu_{i}}\right)}^{1/\alpha }{\left(\frac{\alpha \mu_{i}}{1+\alpha \mu_{i}}\right)}^{y_{i}}\frac{\Gamma (y_{i}+1/\alpha )}{\Gamma (1/\alpha )y_{i}!}$$

where μ_i_ = E_i_ν_i_ are predicted emergency admission totals, and conditional variances are var(Y_i_) = $\underline{\mu_{i}}+{\alpha \mu }_{i}^{2}$. Relative risks of emergency admission ν_i_ are predicted from a log-link regression including only quality of care:

log(ν_i_) = γ_0_ + γ_1_F_i_. (A6)

Model 1 is a joint likelihood over Y and Z data, represented by Equations (A1)–(A6).

In model 2, we additionally consider the impacts on emergency admissions of a socioeconomic deprivation index, D_i_, and also of diabetes morbidity (M_i_). Thus:

Y_i_ ~ NB(E_i_ν_i_,α) (A7)

log(ν_i_) = γ_0_ + γ_1_F_i_ + γ_2_D_i_ + γ_3_M_i_. (A8)

Model 2 also allows for an indirect impact of deprivation on hospitalisations, via quality of care. This leads to a multiple indicator, multiple cause form of structural equation model. Thus with deprivation scores D_i_, centred for identifiability, one has:

Z_ij_ ~ Bin(N_ij_,π_ij_), (A9)

logit(π_ij_) = ς_j_ + λ_j_F_i_ + u_ij_, (A10)

u_ij_ ~ N(0,ψ_j_), (A11)

F_i_ ~ N(β_1_D_i_,θ). (A12)

The joint likelihood under the second model is represented by Equations (A7)–(A12).

In model 3, the latent variable model encompasses both poor access (denoted G) and quality of care (denoted F). Then:

W_ik_ ~ Bin(M_ik_,ξ_ik_), k = 1, …, K (A13)

logit(ξ_ik_) = ζ_j_ + κ_k_G_i_ + e_ik_, (A14)

e_ik_ ~ N(0,ϑ_k_), (A15)

Z_ij_ ~ Bin(N_ij_,π_ij_), j = 1, …, J (A16)

logit(π_ij_) = ς_j_ + λ_j_F_i_ + u_ij_, (A17)

u_ij_ ~ N(0,ψ_j_), (A18)

(F_i_,G_i_) ~ N_2_(μ_i_,Σ), (A19)

(μ_i1_,μ_i2_) = (β_1_D_i_, β_2_D_i_ + β_3_S_1i_ + β_4_S_2i_) (A20)

with N_2_ denoting a bivariate normal density. Causes of poor access are deprivation and supply indicators, as in (A20). For identifiability, κ_1_ = 1, with remaining κ loadings constrained to be positive in line with a poor access interpretation for the G scores. The model for emergency admissions includes access, as well as deprivation and morbidity, in view of evidence such as [8]:

Y_i_ ~ NB(E_i_ν_i_,α) (A21)

log(ν_i_) = γ_0_ + γ_1_F_i_ + γ_2_D_i_ + γ_3_G_i_ + γ_4_M_i_ (A22)

The joint likelihood under the third model is defined by Equations (A13)–(A22). Appendix C contains the code for the bivariate normal model 3.

A bivariate Student t density for quality and access factors is obtained by a scale mixture, namely:

(F_i_,G_i_) ~ N_2_(μ_i_,Σ/ϕ_i_),

ϕ_i_ ~ Gamma(0.5δ,0.5δ),

where δ is the Student’s t degrees of freedom. Following [52] (p. 449), a robust analysis is obtained by taking a preset degrees of freedom δ = 4.

## Appendix B. Prior Densities and Fit Measures

We assume gamma priors, with shape 1 and index 0.01, on inverse variance parameters (1/ψ_j_, 1/ϑ_k_, 1/θ) and the negative binomial parameter 1/α, and Normal priors with mean zero and precision 0.001 on fixed effects (such as regression intercepts and slopes). The precision matrix ∑ in model 3 is assigned a Wishart prior, with identity scale matrix and two degrees of freedom. The unknown loadings λ_j_ and κ_k_ in the factor score models are assigned exponential priors with mean 1. Estimates are based on the second halves of two chain runs of 20,000 iterations, with convergence assessed using Brooks-Gelman-Rubin diagnostics [60].

Fit is assessed using the WAIC information criterion [61], with WAIC obtained only on the Y and Z data in models 1 and model 2, and for the Y, Z, and W data in model 3. Posterior predictive checks [62,63] are also applied. For the emergency admissions model, these are based on predicted emergency admissions Y_new,i_ sampled from the relevant posterior predictive density, namely p(Y_new_|Y,Z) in models 1 and 2, and p(Y_new_|Y,Z,W) in models 3 and 4. Posterior predictive checks for the clinical and access indicators are based on predicted clinical responses Z_new_, and predicted access indicators W_new_.

With R and R_new_ denoting fit measures using observations and predictions respectively, posterior predictive *p*-values are estimated as Pr(R_new_ > R|Y,Z) or Pr(R_new_ > R|Y,Z,W), the proportion of iterations where R_new_ > R. Extreme *p*-values (under 0.05 or over 0.95) indicate model discrepancies. A chi-square fit measure is used, with R_Y_ = ∑_i_(Y_i_−μ_i_)^2^/μ_i_, and R_Y,new_ = ∑_i_(Y_new,i_−μ_i_)^2^/μ_i_ for the Y data. For the clinical indicators, R_Z__j_ = ∑_i_(Z_ij_−N_ij_π_ij_)^2^/[N_ij_π_ij_] and R_Z__j,new_ = ∑_i_(Z_new,ij_−N_ij_π_ij_)^2^/[N_ij_π_ij_]. For the access indicators, R_W__k_ = ∑_i_(W_ik_−M_ik_ξ_ik_)^2^/[M_ik_ξ_ik_] and R_W__k,new_ = ∑_i_(W_new,ik_−M_ik_ξ_ik_)^2^/[M_ik_ξ_ik_].

## Appendix C

The core of the rjags code for the bivariate normal factor model 3 is as follows, with terms to extract WAIC elements and predictive checks excluded for brevity.

# N GP practices; J quality indicators; K access indicators
model {for (i in 1:N) {# outcome model
y[i] ~ dnegbin(p.emrg[i],r)
p.emrg[i] <- r/(r+lambda[i])
lambda[i] <- E[i]*nu[i]
# dep.c, deprivation; mrb.c, diabetes prevalence
log(nu[i]) <- gam0+gam[1]*F[1,i]+gam[2]*dep.c[i] 
                        +gam[3]*F[2,i]+ gam[4]*mrb.c[i]
# factor model (F1 is quality of care, F2 is poor access)
F[1:2,i] ~ dmnorm(mu[1:2,i],tau.F[,])
# dep.c, deprivation; supp.c, GP supply; lst.c, list size
mu[1,i] <- beta[1]*dep.c[i]
mu[2,i] <- beta[2]*dep.c[i] +beta[3]*supp.c[i]+beta[4]*lst.c[i]
# measurement models
for (j in 1:J) {Z[i,j] ~ dbin(pi[i,j],N[i,j])
                               logit(pi[i,j]) <- omeg.pi[j]+lambda[j]*F[1,i]+u[i,j]
                               u[i,j] ~ dnorm(0,tau.u[j])}
for (k in 1:K) {W[i,k] ~ dbin(xi[i,k],M[i,k]) 
                            logit(xi[i,k]) <- omeg.xi[k]+kappa[k]*F[2,i]+e[i,k]
                            e[i,k] ~ dnorm(0,tau.e[k])}}
# priors                 
gam0 ~ dnorm(0,0.001)
for (j in 1:4) {gam[j] ~ dnorm(0,0.001); p.gam[j] <- step(gam[j]); 
                             beta[j] ~ dnorm(0,0.001)}
for (j in 1:J) {omeg.pi[j] ~ dnorm(0,0.001); tau.u[j] ~ dgamma(1,0.01)}
for (k in 1:K) {omeg.xi[k] ~ dnorm(0,0.001); tau.e[k] ~ dgamma(1,0.01)}
r ~ dgamma(1,0.01) ; alpha <- 1/r
lambda[1] <- 1; for (j in 2:J) {lambda[j] ~ dexp(1)}
kappa[1] <- 1;    for (j in 2:K) {kappa[k] ~ dexp(1)}
tau.F[1:2 , 1:2] ~ dwish(S[ , ], 2)
# correlation between factors
     sig.F <- inverse(tau.F); cor.F <- sig.F[1,2]/sqrt(sig.F[1,1]*sig.F[2,2]) }
